# Myoelectric Activity and Fatigue in Low-Load Resistance Exercise With Different Pressure of Blood Flow Restriction: A Systematic Review and Meta-Analysis

**DOI:** 10.3389/fphys.2021.786752

**Published:** 2021-11-22

**Authors:** Victor Sabino de Queiros, Ingrid Martins de França, Robert Trybulski, João Guilherme Vieira, Isis Kelly dos Santos, Gabriel Rodrigues Neto, Michal Wilk, Dihogo Gama de Matos, Wouber Hérickson de Brito Vieira, Jefferson da Silva Novaes, Piotr Makar, Breno Guilherme de Araújo Tinoco Cabral, Paulo Moreira Silva Dantas

**Affiliations:** ^1^Graduate Program in Health Sciences, Federal University of Rio Grande do Norte (UFRN), Natal, Brazil; ^2^Graduate Program in Physiotherapy, Federal University of Rio Grande do Norte (UFRN), Natal, Brazil; ^3^Department of Medical Sciences, The Wojciech Korfanty School of Economics, Katowice, Poland; ^4^Provita Zory Medical Center, Zory, Poland; ^5^Graduate Program in Physical Education, Federal University of Juiz de Fora (UFJF), Juiz de Fora, Brazil; ^6^Graduate Program in Family Health, Faculties of Nursing and Medicine Nova Esperança (FACENE/FAMENE), João Pessoa, Brazil; ^7^Coordination of Physical Education, University Center for Higher Education and Development (CESED/UNIFACISA/FCM/ESAC), Campina Grande, Brazil; ^8^Institute of Sport Sciences, Jerzy Kukuczka Academy of Physical Education in Katowice, Katowice, Poland; ^9^Faculty of Kinesiology and Recreation Management, University of Manitoba, Winnipeg, MB, Canada; ^10^Graduate Program at the School of Physical Education and Sport at the Federal University of Rio de Janeiro (UFRJ), Rio de Janeiro, Brazil; ^11^Faculty of Physical Education, Gdańsk University of Physical Education and Sport, Gdańsk, Poland; ^12^Graduate Program in Physical Education, Federal University of Rio Grande do Norte (UFRN), Natal, Brazil

**Keywords:** KAATSU, vascular occlusion, strength training, metabolic stress, electromyography, muscle excitability, torque, central fatigue

## Abstract

**Background:** Low-load resistance exercise (LL-RE) with blood flow restriction (BFR) promotes increased metabolic response and fatigue, as well as more pronounced myoelectric activity than traditional LL-RE. Some studies have shown that the relative pressure applied during exercise may have an effect on these variables, but existing evidence is contradictory.

**Purpose:** The aim of this study was to systematically review and pool the available evidence on the differences in neuromuscular and metabolic responses at LL-RE with different pressure of BFR.

**Methods:** The systematic review and meta-analysis was reported according to PRISMA items. Searches were performed in the following databases: CINAHL, PubMed, Scopus, SPORTDiscus and Web of Science, until June 15, 2021. Randomized or non-randomized experimental studies that analyzed LL-RE, associated with at least two relative BFR pressures [arterial occlusion pressure (AOP)%], on myoelectric activity, fatigue, or metabolic responses were included. Random-effects meta-analyses were performed for MVC torque (fatigue measure) and myoelectric activity. The quality of evidence was assessed using the PEDro scale.

**Results:** Ten studies were included, all of moderate to high methodological quality. For MVC torque, there were no differences in the comparisons between exercise with 40–50% vs. 80–90% AOP. When analyzing the meta-analysis data, the results indicated differences in comparisons in exercise with 15–20% 1 repetition maximum (1RM), with higher restriction pressure evoking greater MVC torque decline (4 interventions, 73 participants; MD = −5.05 Nm [95%CI = −8.09; −2.01], *p* = 0.001, *I*^2^ = 0%). For myoelectric activity, meta-analyses indicated a difference between exercise with 40% vs. 60% AOP (3 interventions, 38 participants; SMD = 0.47 [95%CI = 0.02; 0.93], *p* = 0.04, *I^2^* = 0%), with higher pressure of restriction causing greater myoelectric activity. This result was not identified in the comparisons between 40% vs. 80% AOP. In analysis of studies that adopted pre-defined repetition schemes, differences were found (4 interventions, 52 participants; SMD = 0.58 [95%CI = 0.11; 1.05], *p* = 0.02, *I*^2^ = 27%).

**Conclusion:** The BFR pressure applied during the LL-RE may affect the magnitude of muscle fatigue and excitability when loads between 15 and 20% of 1RM and predefined repetition protocols (not failure) are prescribed, respectively.

**Systematic Review Registration:** [http://www.crd.york.ac.uk/prospero], identifier [CRD42021229345].

## Introduction

Blood flow restriction (BFR) is a commonly used technique by physical therapists and trainers aiming at physical rehabilitation and neuromuscular adaptations (Nakajima et al., 2006; Patterson and Brandner, 2018; de Queiros et al., 2021). This is certainly justified by the fact that some evidence indicates that low-load {20–40% of 1 repetition maximum [1RM] (Lopez et al., 2021)} resistance training with arterial BFR and venous occlusion artificially induced can promote gains in muscle strength and hypertrophy more pronounced than low-load resistance training without BFR (NO-BFR) (Loenneke et al., 2012) and, in some cases, similar to NO-BFR high-load resistance training (Takarada et al., 2000a; Laurentino et al., 2012). Due to structural and functional adaptations independent of high mechanical stress, BFR resistance training has been recommended for clinical populations with articular limitations for high-load resistance training (Vanwye et al., 2017). In addition, BFR resistance training has been suggested as a training option for athletes seeking to maximize muscle hypertrophy gains (Rolnick and Schoenfeld, 2020).

Low-load resistance exercise with BFR promotes increased blood lactate and intramuscular inorganic phosphate concentrations, and more pronounced intramuscular pH reductions than NO-BFR low-load resistance exercise with equalized training volume (Takarada et al., 2000b; Suga et al., 2009; Sugaya et al., 2011; Wilson et al., 2013). Increased metabolic stress has been postulated as a potential mechanism of muscle hypertrophy arising after BFR resistance training (Loenneke et al., 2011; Jessee et al., 2018a; Rolnick and Schoenfeld, 2020). Accumulation of metabolites appears to accelerate fatigue via stimulation of type III and IV afferent fibers [central fatigue mechanism (Amann et al., 2015)], and to maintain force levels, motor units (MU) of high threshold excitability are recruited, therefore a hypertrophic stimulus would be provided to a greater proportion of muscle fibers (Jessee et al., 2018a). This mechanism has been used to explain the increased myoelectric activity in resistance exercise with BFR, compared to NO-BFR low-load resistance exercise (Rolnick and Schoenfeld, 2020).

Considering a possible association between metabolite-induced fatigue with muscle hypertrophy, some authors have investigated how manipulating the BFR pressure applied during exercise can exert an effect on the level of acute fatigue, measured through of maximum voluntary contraction (MVC) torque analysis (Cook et al., 2007; Yasuda et al., 2009; Counts et al., 2015; Fatela et al., 2016). The results presented so far are divergent. Cook et al. (2007) did not identify differences in the levels of acute fatigue (MVC torque decline) between exercises performed with BFR at a pressure of 160 or 300 mmHg. However, Yasuda et al. (2009), testing the same pressures (i.e., 160 and 300 mmHg), verified that exercise performed with greater occlusion pressure evoked a higher level of acute fatigue. We acknowledge that the studies in question have limitations in the methodology used to generate BFR, given that arbitrary pressures were prescribed. However, this divergence can also be identified among studies that used relative pressures based on arterial occlusion pressure (AOP%) values (Counts et al., 2015; Fatela et al., 2016). Counts et al. (2015) identified no difference in the magnitude of the decrease in MVC torque between exercise performed with BFR at 40 vs. 90% AOP, whereas Fatela et al. (2016) found that a higher occlusion pressure was required (80% vs. 40–60% AOP) for low-load exercise to induce a significant MVC torque decline.

As presented, the studies analyzing the effect of constraint pressure manipulation on neuromuscular fatigue show distinct results and this is possibly justified by the exercise settings [e.g., prescribed repetitions scheme and intensity (1RM%)]. In this regard, a robust meta-analysis assigning appropriate weight to each study as part of an integrative analysis becomes important. Therefore, the aim of the present study was to systematically review and meta-analyze the available evidence on the differences in neuromuscular [myoelectric activity and fatigue, here defined by a MVC torque performance reduction (Vøllestad, 1997)] and metabolic responses between low-load resistance exercise with different relative pressure of blood flow restriction. The results of this systematic review may be useful in understanding the effects of different restriction pressures on neuromuscular responses and assist trainers in more appropriate and safer prescription.

## Methods

This systematic review and meta-analysis was reported according to the preferred reporting item guidelines for systematic reviews and meta-analyses (PRISMA) (Page et al., 2021).

### Eligibility Criteria

In our analysis we considered studies that adopted the following criteria, population: healthy human beings (18–80 years) of both genders, trained or untrained; intervention and comparative: low load (≤ 40% of 1RM) resistance exercise performed with at least two BFR pressures (at different times) induced by pneumatic cuff and relativized based on AOP values; outcomes: MVC torque (used to identify fatigue), myoelectric activity, blood lactate concentrations, intramuscular metabolic changes [metabolite concentrations (e.g., inorganic phosphate) and changes in intramuscular pH]; study design: randomized or non-randomized experimental studies. Reviews, letters to the editor, animal studies, expert opinion, studies that analyzed aerobic exercise, passive restriction (i.e., no exercise), practical BFR protocols (i.e., BFR induced by elastic bandaging) were not considered for analyses.

### Information Sources and Searches

The studies were retrieved from electronic database search and from a comprehensive sweeping in the reference list of the included studies (Horsley et al., 2011). A search was conducted on June 15 2021 in the following databases: Cumulative Index to Nursing and Allied Health Literature (CINAHL - EBSCO), National Library of Medicine (PubMed^®^), Scopus (Elsevier), SPORTDiscus (EBSCO) and Web of Science (Clarivate Analytics).

### Search Strategy

The search strategy combined the following descriptors and Boolean operators (AND/OR): (“blood flow restriction” OR “vascular occlusion” OR KAATSU) AND (“resistance training” OR “strength training” OR “resistance exercise” OR “weightlifting” OR “weight-lifting” OR “weight lifting”) AND (“metabolic stress” OR “lactate” OR “fatigue” OR “muscle activation” OR “torque” OR “maximal voluntary isometric contraction” OR “maximal voluntary contraction”). Full details of these supplementary searches can be found in the additional file.

### Selection Process

The studies were selected by two independent reviewers (VQ and IF). Disagreements between reviewers were resolved by a third reviewer (IKS). The screening of studies was divided into three steps: elimination of duplicates (Step 1), reading of titles and abstracts (Step 2), reading of the full article (Step 3). We used the Rayyan QCRI^®^ application (Ryyan QCRI, Qatar Computing Research Institute, HBKU, Doha, Qatar) (Ouzzani et al., 2016) to assist in eliminating duplicates and screening from titles and abstracts.

### Data Extraction

After reading the full articles, two reviewers (VSQ and IMF) independently performed the data extraction of the included studies, encompassing the prescribed exercise configuration (load, volume, recovery interval), pressures tested, variables analyzed, sample characteristics, and results identified. When results were reported in graphs or were not available in the manuscript, the corresponding author was contacted, via email, to request descriptive data of mean and standard deviations and other relevant information. When data were not available, we used ImageJ software^1^ to extract the information directly from the graphs presented in the manuscript.

### Assessment of the Risk of Bias of the Included Studies

After the literature search and selection, risk of bias assessment was performed independently by two authors (VSQ and IMF) and disagreements were resolved by a third researcher (IKS) using the Physiotherapy Evidence Database (PEDro) scale, which has been shown to be a valid measure of the methodologic quality of randomized trials (Elkins et al., 2010) and displays acceptable inter-rater reliability (Moseley et al., 2002). Thus, scores on PEDro scale ranged from 0 (high risk of bias) to 10 (low risk of bias). The quality of the studies was used for qualitative assessment, and it was not an exclusion criteria. The methodological quality of the study was categorized as follows: a score ranging from 6 to 10 was indicative of high quality; whereas scores of 4–5 indicated moderate quality; and scores ≤3 indicated low quality (Valkenet et al., 2011).

### Data Analysis

Statistical analyses were performed using the Review Manager software, version 5.4 (RevMan 5.4, Cochrane Collaboration). The heterogeneity between the studies was quantified through the I^2^ statistic. The heterogeneity was classified according to the following scale: low (< 25%), moderate (25–49%), and high (> 50%) (Higgins and Thompson, 2002). When data were reported on the same scale, a random effects model was used to analyze the mean difference [MD ± 95% confidence interval (95%CI)] (Ahn and Kang, 2018). For torque analyses, the mean values and standard deviation (SD) of pre- and post-exercise torque were considered. The SD_*change*_ was defined by root square [(SD_*pre*2_/N_*pre*_) + (SD_*post*2_/N_*post*_)] (Borenstein et al., 2021). For the surface electromyography (sEMG) analyses, the mean values and the SD of the last measurement taken were considered. For these analyses, the standardized mean difference (SMD) was considered. Due to the variability of the lower extremity sEMG analyses, the following prioritization order was adopted: vastus medialis > vastus lateralis > rectus femoris. When the study analyzed more than one load (1RM%), the load that most closely matched the other studies was considered. When possible, subgroup analyses were introduced to explore the effects of load and repetition scheme configuration (failure vs. not failure) on the results. It was not possible to analyze publication bias, given that an insufficient number of studies (< 10) were included in our quantitative synthesis (Sterne et al., 2011).

#### Sensitivity Analysis

We replicated all meta-analyses performed with three studies that showed high heterogeneity, but excluding outliers, defined by the magnitude and direction of the effect, that appeared in the analyses.

### Certainty of Evidence

The quality of the evidence was assessed through the Grading of Recommendations Assessment, Development and Evaluation (GRADE) (Atkins et al., 2005). Grading of Recommendations Assessment, Development and Evaluation approach suggests the classification of randomized controlled trials initially as high-quality studies (score 4), that go through the specific risk of bias assessments to identify whether their scores need to be reduced to moderate, low, or very low. The following topics were assessed: (1) quality of the original studies; (2) inconsistency of the results (heterogeneity); (3) indirect evidence; (4) imprecision; and (5) publication bias. One point was removed from the quality of the original studies when 50% of the studies in a determined meta-analysis had 1 item (specified in the Table 1) assessed as high risk. For inconsistency, we remove a point if statistical heterogeneity was found. The risk of indirect evidence was assessed considering three factors: (1) when the participants differed from the population of interest; (2) when the interventions differed from the specific desired intervention; and (3) when substitute outcomes were used instead of the relevant ones. The imprecision was assessed based on the total sample size < 100 participants. Regarding publication bias, we did not perform any analysis.

**TABLE 1:** Methodological quality of the included studies.

Study	1	2	3	4	5	6	7	8	9	10	11	Total (0–10)
Counts et al. (2015)	—	+	−	+	−	−	−	+	+	+	+	6
Loenneke et al. (2015)	—	+	−	+	−	−	−	+	+	+	+	6
Loenneke et al. (2016)	—	+	−	+	−	−	+	+	+	+	+	7
Fatela et al. (2016)	—	+	−	+	−	−	−	+	+	+	+	6
Jessee et al. (2017)	—	+	−	+	−	−	−	+	+	+	+	6
Dankel et al. (2017)	—	+	−	+	−	−	−	+	+	+	+	6
Buckner et al. (2018)	—	+	−	+	−	−	−	+	+	+	+	6
Jessee et al. (2019)	—	+	−	NR	−	−	−	+	+	+	+	5
Ilett et al. (2019)	—	+	−	+	−	−	−	+	+	+	+	6
Singer et al. (2020)	—	−	−	+	−	−	−	+	+	+	+	5

*1 – Eligibility criteria specified; 2 – Random allocation; 3 – Concealed allocation; 4 – Groups similar at Baseline; 5 – Participant blinding; 6 – Therapist blinding; 7 – Assessor blinding; 8 – Adequate follow-up; 9 – Intention to-treat analysis; 10 - Between group comparisons; 11 – Point estimates and variability; NR: not reported.*

## Results

### Study Selection

**FIGURE 1:**
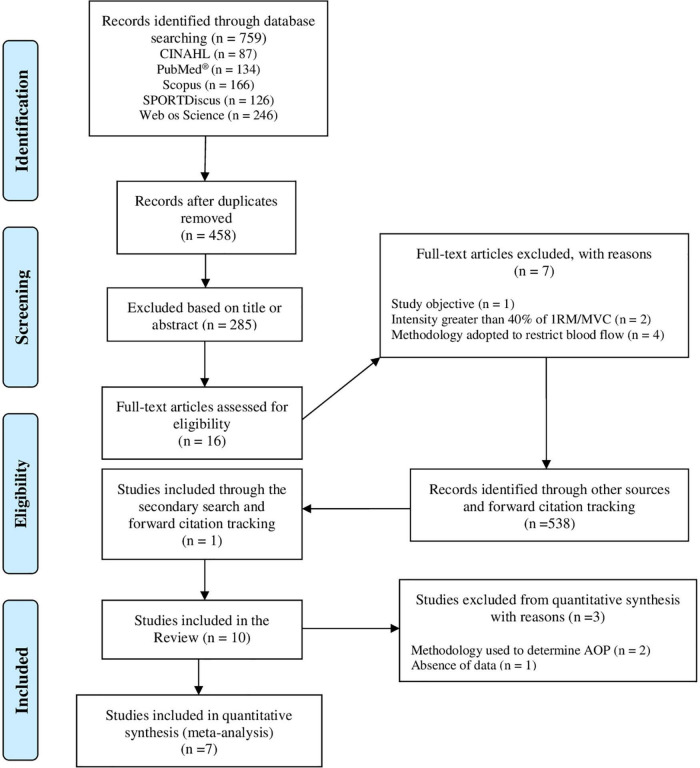
PRISMA flow diagram of study selection.

A total of 759 studies were identified in the databases. Ten studies were included in this review and seven studies were included in the meta-analysis. The flow diagram of the literature search is presented in Figure 1.

### Study Characteristics

Eighty percent (80%) of the included studies adopted a crossover design (Counts et al., 2015; Fatela et al., 2016; Dankel et al., 2017; Jessee et al., 2017, 2019; Buckner et al., 2018; Ilett et al., 2019; Singer et al., 2020) and 20% of the studies used a parallel design (independent groups) (Loenneke et al., 2015, 2016). The studies encompassed a total of 174 participants, with a mean age ranging from 22 to 25 years. Two studies analyzed the same sample (Loenneke et al., 2015, 2016), so only the sample number reported in one of the studies in question was counted. Seventy percent (70%) of the studies analyzed trained individuals (*n* = 139) (Counts et al., 2015; Loenneke et al., 2015, 2016; Dankel et al., 2017; Jessee et al., 2017, 2019; Buckner et al., 2018) and 30% of the studies considered untrained individuals (*n* = 35) (Fatela et al., 2016; Ilett et al., 2019; Singer et al., 2020). All studies analyzed a single-joint exercise (knee extension, *n* = 6; elbow flexion, *n* = 4). Thirty percent (30%) of the studies analyzed more than one intensity (1RM%) (Loenneke et al., 2015, 2016; Dankel et al., 2017). The characteristics of the participants and studies are presented in detail in Tables 2, 3.

**TABLE 2:** Characteristics of the participants.

Study	Participants (*n* = 174)	Age (years)	Height (cm)	Body mass (kg)	Training status
Counts et al. (2015)	14	24.0 ± 3.0	174.0 ± 6.7	79.7 ± 11.3	Trained
Loenneke et al. (2015)	40	22.3 ± 3.6	176.6 ± 6.3	81.9 ± 13.2	Trained
Loenneke et al. (2016)	40	22.3 ± 3.6	176.6 ± 6.3	81.9 ± 13.2	Trained
Fatela et al. (2016)	14	24.8 ± 5.4	175.2 ± 4.4	71.1 ± 6.9	Untrained
Jessee et al. (2017)	26	22.0 ± 2.6	175.3 ± 10.6	78.7 ± 14.0	Trained
Dankel et al. (2017)	14	24.0 ± 3.8	175.0 ± 11.4	83.0 ± 17.1	Trained
Buckner et al. (2018)	22	22.0 ± 2.0	174.7 ± 10.4	76.0 ± 17.0	Trained
Jessee et al. (2019)	23	22.0 ± 2.7	174.5 ± 10.2	75.7 ± 17.3	Trained
Ilett et al. (2019)	10	25.0 ± 6.0	176.8 ± 5.6	78.1 ± 8.5	Untrained
Singer et al. (2020)	11	25.0 ± 4.0	Not reported	77.8 ± 7.9	Untrained
**Mean ± SD**	21.4 ± 11.1	23.0 ± 3.7	175.2 ± 8.2	78.4 ± 13.9	—
**Range**	40.0 – 10.0	25.0 – 22.0	176.8 – 174.0	83.0 – 75.7	—

*n = sample size; SD = standard deviation.*

**TABLE 3:** Summary and characteristics of the studies included in the review.

Study (Year)	Study design	Impact factor	Resistance exercise	Exercise protocol	AOP% (Cuff size)	Outcome measure (s)
Counts et al. (2015)	Crossover	2.5	Elbow flexion	**LL:** 75 reps (30-15-15-15); @30% of 1RM; 30s interval between sets.	40, 50, 60, 70, 80, 90% (5cm)	MVC isometric torque (Nm), sEMG Amplitude (RMS).
Loenneke et al. (2015)	Parallel	2.5	Knee extension	**LL (BFR):** 75 reps (30-15-15-15); @20% and 30% de 1RM; 30s interval between sets. **LL:** 4 sets of muscle failure; @20 and 30% of 1RM; 30s interval between sets. **HL:** 4 sets of 10 reps; @70% of 1RM; 60s interval between sets.	0,40, 50, 60% (5cm)	MVC isometric torque (Nm), sEMG Amplitude (RMS).
Loenneke et al. (2016)	Parallel	1.7	Knee extension	**LL (BFR):** 75 reps (30-15-15-15); @20% and 30% de 1RM; 30s interval between sets. **LL:** 4 sets of muscle failure; @20 and 30% of 1RM; 30s interval between sets. **HL:** 4 sets of 10 reps; @70% of 1RM; 60s interval between sets.	0,40, 50, 60% (5 cm)	Blood lactate (mmol^–1^)
Fatela et al. (2016)	Crossover	2.9	Knee extension	**LL:** 75 reps (30-15-15-15); @20% of 1RM; 30s interval between sets.	40, 60, 80% (13 cm)	MVC isometric torque (Nm), sEMG Amplitude (RMS)
Jessee et al. (2017)	Crossover	1.7	Elbow flexion	**LL:** 75 reps (30-15-15-15); @30% of 1RM; 30s interval between sets	0,10,20,30,50, 90% (5 cm)	MVC isometric torque (Nm), sEMG Amplitude (RMS).
Dankel et al. (2017)	Crossover	1.4	Elbow flexion	**LL:** 75 reps (30-15-15-15); @10%, 15% and 20% of 1RM; 30s interval between sets	40, 80% (5cm)	MVC isometric torque (Nm), sEMG Amplitude (RMS).
Buckner et al. (2018))	Crossover	1.7	Elbow flexion	**LL:** 4 sets of muscle failure; @15% of 1RM; 30s interval between sets. **HL:** 4 sets of muscle failure; @70% of 1RM; 90s interval between sets.	0,40, 80% (5 cm)	MVC isometric torque (Nm), sEMG Amplitude (RMS).
Jessee et al. (2019)	Crossover	1.4	Knee extension	**LL:** 4 sets of muscle failure; @15% of 1RM; 30s interval between sets. **HL:** 4 sets of muscle failure; @70% of 1RM; 90s interval between sets.	0,40, 80% (10 cm)	MVC isometric torque (Nm), sEMG Amplitude (RMS).
Ilett et al. (2019)	Crossover	4.1	Knee extension	**LL:** 75 reps (30-15-15-15); 20% of 1RM; 30s interval between sets. **HL:** 4 sets of 8 reps; @80% 1RM; 150s interval between sets.	0,40, 60, 80% (10.5 cm)	MVC isometric torque (Nm), sEMG Amplitude (RMS), Blood lactate (mmol/L).
Singer et al. (2020)	Crossover	2.9	Knee extension	**LL:** 30 reps (1 set); @30% of peak torque.	0,60, 80, 100% (10 cm)	sEMG Amplitude (RMS)

*RM: repetition maximum; AOP: arterial occlusion pressure; BFR: blood flow restriction; sEMG: surface electromyography; HL: high load; LL: low load; MVC: maximum voluntary contraction; RMS: root mean square; @: load used.*

### Determination of Blood Flow Restriction Pressure

Seventy percent (70%) of studies determined AOP directly using a vascular Doppler (Counts et al., 2015; Fatela et al., 2016; Dankel et al., 2017; Jessee et al., 2017, 2019; Buckner et al., 2018; Singer et al., 2020), while 10% of studies used an automated tourniquet to determine AOP (Ilett et al., 2019) and 20% of studies estimated AOP from limb circumference (Loenneke et al., 2015, 2016). Sixty percent (60%) of the studies assessed AOP before each exercise session (Dankel et al., 2017; Jessee et al., 2017, 2019; Buckner et al., 2018; Ilett et al., 2019; Singer et al., 2020) and 20% of the studies performed a single measurement on a different day from the experimental session (Counts et al., 2015; Fatela et al., 2016). Sixty percent (60%) of the studies specified that the measurement was performed in the exercise position (Counts et al., 2015; Dankel et al., 2017; Jessee et al., 2017, 2019; Buckner et al., 2018; Singer et al., 2020), while 20% of the studies offered no details about the position adopted for AOP assessments (Fatela et al., 2016; Ilett et al., 2019).

### Methodological Quality (Internal Validity)

Eighty percent (80%) of trials received a score between 6 and 10 on the PEDro scale (high methodological quality), while 20% of trials received a score of 5 (moderate methodological quality). Only one study reported blinding of outcome assessors (Loenneke et al., 2016). A single study did not report implementation of randomization (Singer et al., 2020). Although most studies reported use of randomization, none of the studies offered details on how this procedure was performed. In addition, none of the studies reported the existence of a record of the research protocol. The quality ratings for each study included in the review are presented in Table 1.

### Main Outcomes

#### Maximum Voluntary Contraction Isometric Torque (Fatigue)

**FIGURE 2:**
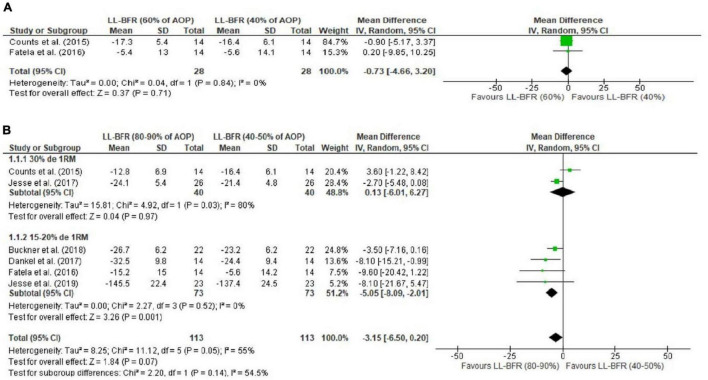
Forest plot illustrating the combined effects for touch MVC torque of: **(A)** LL-RE with BFR pressure of 40% AOP vs. RE-LL with BFR pressure of 60% AOP; **(B)** LL-RE with BFR pressure of 40–50% AOP vs. LL-RE with BFR pressure of 80–90% AOP. 1RM: 1 repetition maximum; 95%CI: Confidence interval; AOP: arterial occlusion pressure; LL-BFR: low load + blood flow restriction; LL: low load; SD: standard deviation.

**FIGURE 3:**
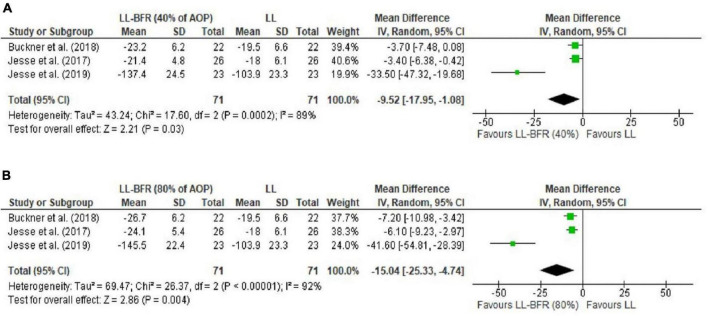
Forest plot illustrating the combined effects for touch MVC of: **(A)** LL-RE with BFR pressure of 40% AOP vs. LL-RE (NO-BFR); **(B)** LL-RE with BFR pressure of 80% AOP vs. LL-RE (NO-BFR). 95%CI: confidence interval; AOP: arterial occlusion pressure; LL-BFR: low load + blood flow restriction; LL: low load; SD: standard deviation.

The meta-analysis performed for MVC isometric torque indicated no difference for 40% vs. 60% AOP (MD = −0.73 Nm [95CI% = −4.66; 3.20]; *p* = 0.71; *I*^2^ = 0%) (Figure 2A). A trend was identified in comparisons of 40–50% vs. 80–90% AOP (MD = −3.15 Nm [95%CI = −6.50; 0.20]; *p* = 0.07; *I*^2^ = 55%); subgroup analyses identified that at 15–20% 1RM loading, application of 80-90% AOP promotes greater decrease in torque (MD = − 5.05 Nm [95%CI = −8.09; −2.01]; *p* = 0.001; *I*^2^ = 0%), results that were not observed in exercise at 30% 1RM (MD = 0.13 Nm [95%CI = −6.01; −6.27]; p = 0.97; *I*^2^ = 80%) (Figure 2B). Additionally, exercise with 40–50% AOP or 80–90% AOP was found to induce a greater decrease in isometric torque of the MVC compared to low-load exercise without BFR (0%AOP) (MD = −9.52 Nm [95%CI = −17.95, −1.08]; p = 0.03; *I*^2^ = 89% and MD = −15.04 Nm [95%CI = −25.33; −4.74]; p = 0.004; *I*^2^ = 92%, for exercise with 40–50% and 80–90% AOP, respectively) (Figure 3). Details of the GRADE certainty of evidence classification for the analyses in question are reported in detail in Table 4.

**TABLE 4:** Certainty of evidence according to the GRADE.

Outcome	Comparison (%AOP)	Certainty assessment						No of patients		Effect	Certainty
				
		N° of studies	Study design	Risk of bias	Inconsistency	Indirect evidence	Imprecision	group	group	Absolute (95% CI)	
MVC torque	40% vs. 60%	2	Crossover randomized trials	Serious ^a^	Not serious	Not serious	Serious ^c^	28	28	−4.66 to 3.20	⊕⊕○ LOW
MVC torque	40% vs. 80% (15–20% 1RM)	4	Crossover randomized trials	Serious ^a^	Not serious	Not serious	Serious ^c^	73	73	−8.09 to −2.01	⊕⊕○ LOW
MVC torque	40–50% vs. 80–90% (30% 1RM)	2	Crossover randomized trials	Serious ^a^	Serious ^b^	Not serious	Serious ^c^	40	40	−6.01 to 6.27	⊕○○ VERY LOW
MVC torque	40% vs. 0%	3	Crossover randomized trials	Serious ^a^	Serious ^b^	Not serious	Serious ^c^	71	71	−17.95 to −1.08	⊕○○ VERY LOW
MVC torque	80% vs. 0%	3	Crossover randomized trials	Serious ^a^	Serious ^b^	Not serious	Serious^c^	71	71	−25.33 to −4.74	⊕○○ VERY LOW
EMG	40% vs. 60%	3	Crossover randomized trials	Serious ^a^	Not serious	Not serious	Serious^c^	38	38	0.02 to 0.93	⊕⊕○ LOW
EMG	40% vs. 80% (Not failure)	4	Crossover randomized trials	Serious ^a^	Not serious	Not serious	Serious^c^	52	52	0.11 to 1.05	⊕⊕○ LOW
EMG	40% vs. 80% (Failure)	2	Crossover randomized trials	Serious ^a^	Not serious	Not serious	Serious^c^	40	40	−0.75 to 0.13	⊕⊕○ LOW
EMG	40% vs.0% (Low load)	3	Crossover randomized trials	Serious ^a^	Not serious	Not serious	Serious ^c^	50	50	−0.34 to 0.44	⊕⊕○ LOW
EMG	40% vs. 0% (High load)	3	Crossover randomized trials	Serious ^a^	Serious ^b^	Not serious	Serious ^c^	50	50	−2.37 to −0.01	⊕○○ VERY LOW
EMG	80% vs. 0% (Low load)	3	Crossover randomized trials	Serious ^a^	Serious ^b^	Not serious	Serious^c^	50	50	−0.75 to 0.74	⊕○○ VERY LOW
EMG	80% vs. 0% (High load)	3	Crossover randomized trials	Serious ^a^	Not serious	Not serious	Serious ^c^	50	50	−1.20 to −0.38	⊕⊕○ LOW

*AOP: Arterial occlusion pressure; CI: Confidence interval. Explanations:*

*^a^Lack of blinding of the result evaluators; Lack of details about the randomization process; Absence of study registration.*

*^b^High and significant heterogeneity.*

*^c^Sample size less than 100 for each group.*

##### Sensitivity Analysis

Because of the high heterogeneity identified, sensitivity analyses were implemented for the following comparisons: (i) 40–50% vs. 0% AOP; (ii) 80–90% vs. 0% AOP. After removing one (Buckner et al., 2018) of the three included studies, results remained significant for the comparisons between 80 and 90% vs. 0% AOP (MD = −6.55 Nm [95%CI = −8.96; −4.13]; *p* < 0.00001; *I*^2^ = 0%) and 40-50% vs. 0% of AOP (MD = −3.52 Nm [95%CI = −5.86; −1.17]; *p* = 0.003; *I*^2^ = 0%), but heterogeneity was reduced.

#### Surface Electromyography

**FIGURE 4:**
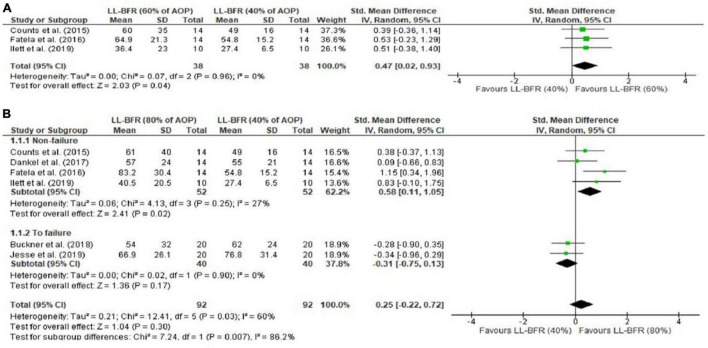
Forest plot illustrating the combined effects for myoelectric activity of: **(A)** LL-RE with BFR pressure of 40% AOP vs. LL-RE with BFR pressure of 60% AOP; **(B)** LL-RE with BFR pressure of 40 AOP vs. LL-RE with BFR pressure of 80% AOP. 95%CI: confidence interval; AOP: arterial occlusion pressure; LL-BFR: low load + blood flow restriction; LL: low load; SD: standard deviation.

**FIGURE 5:**
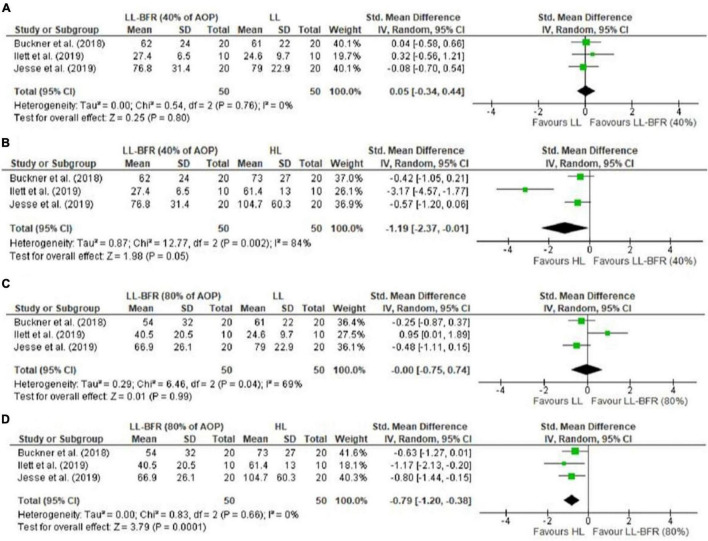
Forest plot illustrating the combined effects for myoelectric activity of: **(A)** LL-RE with BFR pressure of 40% AOP vs. LL-RE (NO-BFR); **(B)** LL-RE with BFR pressure of 40% AOP vs. HL-RE (NO-BFR); **(C)** LL-RE with BFR pressure of 80% AOP vs. LL-RE (NO-BFR); **(D)** LL-RE with BFR pressure of 80% AOP vs. HL-RE (NO-BFR). 95%CI: confidence interval; AOP: arterial occlusion pressure; HL: high load; LL-BFR: low load + blood flow restriction; LL: low load; SD: standard deviation.

Electromyography (sEMG) analyses indicated a difference for 40% vs. 60% AOP, in favor of higher pressures (SMD = 0.47 [95%CI = 0.02; 0.93]; *p* = 0.04; *I*^2^ = 0%) (Figure 4A). This result was not identified for 40% vs. 80% AOP (SMD = 0.25 [95%CI = −0.22; 0.72]; *p* = 0.30; *I*^2^ = 60%); subgroup analyses indicated a difference in these analyses in favor of higher pressures performed not to failure (SMD = 0.58 [95%CI = 0.11; 1.05]; *p* = 0.02; *I*^2^ = 27%) (Figure 4B). We identified no differences for exercise with 40% AOP vs. traditional low-load exercise (SMD = 0.05 [95%CI = −0.34; 0.44]; *p* = 0.80; I^2^ = 0%) (Figure 5A) or exercise with 80% AOP vs. traditional low-load exercise (SMD = −0.00 [95%CI = −0.75; 0.74]; *p* = 0.99; I^2^ = 69%) (Figure 5C). In contrast, differences were identified for exercise with 40% AOP vs. high-load exercise (SMD = −1.19 [95%CI = −2.37; −0.01]; *p* = 0.05; *I*^2^ = 84%) (Figure 5B), and exercise with 80% AOP (SMD = −0.79 [95%CI = −1.20; −0.38]; *p* = 0.0001; *I*^2^ = 0%) (Figure 5D). Details of the GRADE certainty of evidence classification for the analyses in question are reported in detail in Table 4.

##### Sensitivity Analysis

Sensitivity analyses were performed for the following comparisons: (i) exercise with 80% AOP vs. traditional low-load exercise; (ii) exercise with 40% AOP vs. high-load exercise. After we removed one (Buckner et al., 2018) of the three studies included in the analyses, the results remained significant, but heterogeneity was reduced in the comparisons between exercise with 40% AOP vs. high-load exercise (SMD = −0.49 [95%CI = −0.94; −0.05]; *p* = 0.03; *I*^2^ = 0%). Similarly, after we removed one (Buckner et al., 2018) of the three studies included in the analyses, the results remained non-significant, but heterogeneity was reduced in the comparisons between exercise with 80% AOP vs. traditional low-load exercise (SMD = −0.37 [95%CI = −0.81; 0.08]; *p* = 0.11; *I*^2^ = 0%).

## Discussion

The purpose of this systematic review and meta-analysis was to analyze neuromuscular responses reported in low-load resistance exercise combined with different BFR pressures (%AOP). With respect to MVC torque decline (fatigue measure), analyses indicated no difference for 40% vs. 60% AOP. A tendency (*p* = 0.07) was identified for the 40-50% vs. 80–90% AOP comparisons; subgroup analyses indicated that higher pressures (i.e., 80–90% vs. 40–50% AOP) induce more fatigue (MVC torque decline) in exercise at 15–20% of 1RM, but not in exercise at 30% of 1RM. Additionally, exercise at 40–50% or 80–90% appeared to induce more fatigue than traditional low-load exercise (i.e., no BFR; 0% AOP). Regarding sEMG analyses, significant differences were found for 40% vs. 60% AOP and 40% vs. 80% AOP (not to failure) in favor of higher pressures. This result was also identified in the analyses for 40% AOP vs. high-load exercise and 80% AOP vs. NO-BFR high-load resistance exercise, but the results were favorable for NO-BFR high-load resistance exercise.

### Maximum Voluntary Contraction Isometric Torque (Fatigue)

Previous studies have found that the application of BFR during low-load exercise maximizes intramuscular metabolic stress (Suga et al., 2009; Sugaya et al., 2011; Yanagisawa and Sanomura, 2017). To illustrate this, Suga et al. (2009) found that low-load (20% of 1RM) plantar flexion exercise with BFR promotes more pronounced inorganic phosphate accumulation and intramuscular pH reductions than NO-BFR low-load exercise and similar to NO-BFR high-load exercise. The accumulation of metabolites may compromise the contractile capacity of skeletal muscle through metabolic stimulation of group III and IV afferents (mechanoreceptors and metaboreceptors, respectively) and, consequently, reduced motoneuron activity (central mechanism) (Boyas and Guével, 2011). Therefore, amplified metabolite accumulation may be a valid justification to explain, at least in part, the more pronounced MVC torque decline in low-load exercise with BFR, relative to NO-BFR low-load exercise. It is worth noting that these results were identified for the analyses of moderate (40–50% AOP) and high (80–90% AOP) BFR pressure.

Previously, a dose-dependent relationship was found between the restriction pressure applied in exercise and intramuscular metabolite accumulation (Sugaya et al., 2011). In this sense, it was expected that exercise with higher relative BFR pressure would induce greater MVC torque decline. Our analyses indicated no difference for 40% vs. 60% AOP. It is possible that an increase from 40 to 60% AOP is not sufficient to induce some sort of metabolic change that amplifies the magnitude of fatigue. The individual findings of Ilett et al. (2019) support this hypothesis, given that the authors found no differences between MVC torque values assessed over multiple knee extension sets for exercise with BFR at pressure 40% and 60% AOP. Additionally, Loenneke et al. (2016) identified that exercise performed at 40% and 60% of the predicted AOP produces similar acute blood lactate changes, i.e., a similar metabolic response.

Our analyses point to a difference in the decline in MVC torque between exercise with BFR at pressure 40–50% vs. 80–90% of AOP, but only for those studies that analyzed exercise with load 15–20% of 1RM. This finding provides evidence that exercise with loads ≤ 20% of 1RM may be more influenced by manipulation of constraint pressure, with higher%AOP inducing higher levels of fatigue. In the case of studies with loads ≥ 30% of 1RM, it is possible that the applied load itself is sufficient to significantly limit blood flow and therefore the possible effects of constraint pressure manipulation are mitigated Lixandrão et al. (2015). This aspect was used to justify the findings of Lixandrão et al. (2015); the authors found that femoral quadriceps hypertrophy resulting from a 20% 1RM resistance training program is maximized by applying higher BFR pressures (80% vs. 40% AOP), but this dose-dependent effect was not evidenced during resistance training program with load 40% 1RM. Similarly, Counts et al. (2015) did not identify an effect of restriction pressure (40% vs. 90% AOP) on elbow flexor hypertrophy resulting after resistance training program with 30% 1RM load.

### Surface Electromyography

A recent meta-analysis identified that under conditions of equalized volume (not failure), myoelectric activity is greater in low-load exercise with BFR than in traditional low-load exercise (Cerqueira et al., 2021). In contrast, in protocols consisting of sets performed to muscle failure this superiority effect of exercise with BFR was non-existent (Cerqueira et al., 2021). Similarly, we identified that in protocols of preset repetition schemes (not failure), muscle excitability is increased by higher BFR pressures (40% vs. 60%; 40% vs. 80% AOP), but this effect was not identified in protocols of repetitions performed to muscle failure. Taken together, these data suggest that the effect of BFR pressure (e.g., 0 vs. 40% vs. 80% AOP) on muscle excitability disappears in protocols performed to muscle failure. This aspect could explain the fact that we did not find differences in the comparisons made for low-load exercise with BFR (40% or 80% AOP) vs. traditional low-load exercise, since most of the studies included in these analyses analyzed sets up to muscle failure. Thus, it is likely that in sets performed to muscle failure, the stimulus provided by exercise with BFR is similar to the stimulus provided by traditional low-load exercise. Perhaps because of this, Jessee et al. (2018b) did not show an effect of BFR (40% or 80% AOP) on the hypertrophic adaptations provided by a low-load RT program (15% of 1RM) composed of exercise performed to muscle failure.

Although the aforementioned theory finds support to some extent, one needs to consider that in the study by Laurentino et al. (2012), a low-load resistance training program combined with BFR promoted hypertrophy similar to a high-load resistance training program (80% of 1RM). Our analyses indicated that the NO-BFR high-load exercise promoted higher myoelectric activity than the low-load exercise with BFR, regardless of the level of restriction (40% or 80% AOP). These results are in line with the results reported by Cerqueira et al. (2021). Considering a primary role of MU recruitment in the hypertrophic adaptations provided by BFR resistance training, one would expect that NO-BFR high-load resistance training programs would induce more hypertrophy than BFR low-load resistance training programs, since sEMG amplitude is lower in the latter. However, two previously published meta-analyses identified that muscle hypertrophy gain is similar between the training models in question (Lixandrão et al., 2018; Centner et al., 2019). Therefore, acute sEMG data may not be good predictors of muscle hypertrophy. Furthermore, sEMG results reported in BFR exercise studies should be interpreted with some level of caution, due to the fact that sEMG amplitude may not necessarily reflect increased MU recruitment (Vigotsky et al., 2018). Finally, we do not rule out the possibility that other mechanisms (e.g., edema) may be involved in the adaptations provided by BFR low-load resistance training, and not just an increase in MU recruitment.

### Future Research

Under certain conditions (≤ 20% of 1RM), it has been found that increasing the relative pressure of BFR can maximize fatigue, which in theory may be positive for induction of hypertrophic adaptations arising during and after BFR resistance training. However, these findings may be limited for exercise protocols with continuous restriction. In this type of prescription, the restriction pressure is maintained throughout the exercise, including the recovery intervals between sets. We believe that it would be important for future studies to analyze the effect of modulating the pressure of restraint in intermittent restraint protocols (Wilk et al., 2020), characterized by the release of the restraint pressure during recovery intervals and compare it to continuous restraint models. Additionally, we recommend that future studies analyze the acute effect of restraint pressure modulation in repetition schemes consisting of sets of 15 repetitions. This arbitrary repetition scheme may be a more viable option for studies that propose to analyze no-fail conditions, relative to the protocol consisting of 75 repetitions (30-15-15-15). Finally, we recommend that future studies look for more suitable methods to analyze MU recruitment in exercise protocols with BFR, given that surface EMG presents certain limitations for this type of analysis (Vigotsky et al., 2018), since the amplitude of the sEMG is not only dependent on the recruitment of MU, but also on the firing rate and synchronization of all active muscles fibers under electrode area (Lixandrão et al., 2018).

### Limitation

Some aspects should be pointed out for a better understanding of our results. A high heterogeneity was identified for comparisons of torque decline and sEMG between exercise at performed with BFR a pressure 40-50% vs. 80-90% AOP (*I*^2^ = 55% and *I*^2^ = 60% for torque and EMG, respectively), suggesting a certain degree of variability among the studies included in these meta-analyses. However, subgroup analyses were performed in order to isolate the differences and identify possible factors that could account for the different effects. For the torque analyses, a high heterogeneity (*I*^2^ = 80%) was evidenced in the subgroup analyses of the studies that adopted 30% of 1RM load; due to the low number of studies (*n* = 2), we did not perform sensitivity analyses, so these data should be analyzed with caution. Additionally, we identified high heterogeneity among the comparisons performed for exercise with BFR vs. traditional exercise (high-load and low-load). However, we should point out that the results were maintained after our sensitivity analyses.

We pointed out that the quality of evidence was low for all analyses and that the studies had certain important methodological limitations, including lack of blinding of outcome assessors, information about the procedure used for randomization or concealment of this procedure, and information about study registration.

### Practical Application

This review provides important information on how the manipulation of BFR pressure (individualized) applied in low-load exercise can affect fatigue. Therefore, our findings can be used to assist trainers and physical therapists in prescribing resistance training with BFR. In particular, we found that exercise with moderate pressures (40–50% AOP) induces a higher level of fatigue than NO-BFR exercise. Therefore, this BFR pressure may be sufficient to induce an adequate stimulus that reflects significant chronic adaptations, in addition to promoting reduced levels of discomfort, compared to higher BFR pressures (Soligon et al., 2018). We point out that, in the case of acute fatigue, exercise with higher loads (30% of 1RM) seems not to be affected by the manipulation of the BFR pressure. Therefore, the application of high pressures may be unnecessary when the exercise is performed with loads between 30 and 40% of 1RM. This aspect could justify the fact that chronic studies (Counts et al., 2015; Lixandrão et al., 2015) that compared resistance training programs combined with different levels of BFR, applying loads of 30 and 40% of 1RM, did not identify the effect of BFR pressure on muscle hypertrophy.

## Conclusion

In conclusion, the results of this systematic review and meta-analysis demonstrate that low-load resistance exercise with moderate (40–50% AOP) or high (80–90% AOP) blood flow restriction pressure induces more fatigue (decline in neuromuscular function) than NO-BRF low-load resistance exercise. However, applying a high restriction pressure can increase the magnitude of fatigue when loads of 15–20% of 1RM are prescribed. Additionally, we identified that the application of higher restriction pressures can increase muscle excitability in pre-defined repetition schemes (not failure). However, the level of excitability achieved with low-load exercise with moderate or high restriction pressures (40% and 80% AOP, respectively) is still lower than in NO-BFR high-load resistance exercise.

## Data Availability Statement

The original contributions presented in the study are included in the article/Supplementary Material, further inquiries can be directed to the corresponding authors.

## Author Contributions

VQ was responsible for the original idea of the review and wrote the original draft of the manuscript. VQ, IS, GN, BC, and PD were responsible for the study design. VQ and IF performed out the bibliographical research. VQ, IF, and IS participated in the process of screening and evaluating the methodological quality of the studies included in the review. All authors read, critically reviewed, and approved the final version of the manuscript.

## Conflict of Interest

The authors declare that the research was conducted in the absence of any commercial or financial relationships that could be construed as a potential conflict of interest.

## Publisher’s Note

All claims expressed in this article are solely those of the authors and do not necessarily represent those of their affiliated organizations, or those of the publisher, the editors and the reviewers. Any product that may be evaluated in this article, or claim that may be made by its manufacturer, is not guaranteed or endorsed by the publisher.

## Footnotes

1
https://imagej.nih.gov/ij/

## Abbreviations

1RM: 1 repetition maximum
AOP: arterial occlusion pressure
BFR: blood flow restriction
EMG: electromyography
MVC: maximum voluntary contraction
MU: motor units.
