# Supplementary material for: Myoelectric Activity and Fatigue in Low-Load Resistance Exercise With Different Pressure of Blood Flow Restriction: A Systematic Review and Meta-Analysis
Source: Front Physiol. 2021 Nov 22;12:786752. doi: 10.3389/fphys.2021.786752 (PMC8646757; doi:10.3389/fphys.2021.786752)
Supplement: Supplementary file 1 [file Data_Sheet_1.docx]

Supplementary Material

# Supplementary Data

Cumulative Index to Nursing and Allied Health **(CINAHL)**

Updated to June 2021

Filters: Language english; Year of publication (1990-2021).

Results: 87

Keywords and Boolean operators: ("blood flow restriction" OR "vascular occlusion" OR "Kaatsu") AND ("resistance training" OR "strength training" OR "resistance exercise" OR "weightlifting" OR "weight-lifting" OR "weight lifting") AND ("metabolic stress" OR "lactate" OR "fatigue" OR "muscle activation" OR "torque" OR "maximal voluntary isometric contraction" OR "maximal voluntary contraction")

**PubMed^®^**

Updated to June 2021

Filters: Language english; Species Humans.

Results: 134

Keywords and Boolean operators: ("blood flow restriction" OR "vascular occlusion" OR "Kaatsu") AND ("resistance training" OR "strength training" OR "resistance exercise" OR "weightlifting" OR "weight-lifting" OR "weight lifting") AND ("metabolic stress" OR "lactate" OR "fatigue" OR "muscle activation" OR "torque" OR "maximal voluntary isometric contraction" OR "maximal voluntary contraction")

**Scopus**

Updated to June 2021

Filters: Language english; Articles.

Results: 166

Keywords and Boolean operators: ("blood flow restriction" OR "vascular occlusion" OR "Kaatsu") AND ("resistance training" OR "strength training" OR "resistance exercise" OR "weightlifting" OR "weight-lifting" OR "weight lifting") AND ("metabolic stress" OR "lactate" OR "fatigue" OR "muscle activation" OR "torque" OR "maximal voluntary isometric contraction" OR "maximal voluntary contraction")

**SPORTDiscus**

Updated to June 2021

Filters: Language english.

Results: 126

Keywords and Boolean operators: ("blood flow restriction" OR "vascular occlusion" OR "Kaatsu") AND ("resistance training" OR "strength training" OR "resistance exercise" OR "weightlifting" OR "weight-lifting" OR "weight lifting") AND ("metabolic stress" OR "lactate" OR "fatigue" OR "muscle activation" OR "torque" OR "maximal voluntary isometric contraction" OR "maximal voluntary contraction")

**Web of Science**

Updated to June 2021

Filters: Language English; Articles; Year of publication (1990-2021).

Results: 246

Keywords and Boolean operators: ("blood flow restriction" OR "vascular occlusion" OR "Kaatsu") AND ("resistance training" OR "strength training" OR "resistance exercise" OR "weightlifting" OR "weight-lifting" OR "weight lifting") AND ("metabolic stress" OR "lactate" OR "fatigue" OR "muscle activation" OR "torque" OR "maximal voluntary isometric contraction" OR "maximal voluntary contraction")

# Supplementary Figures and Tables

##
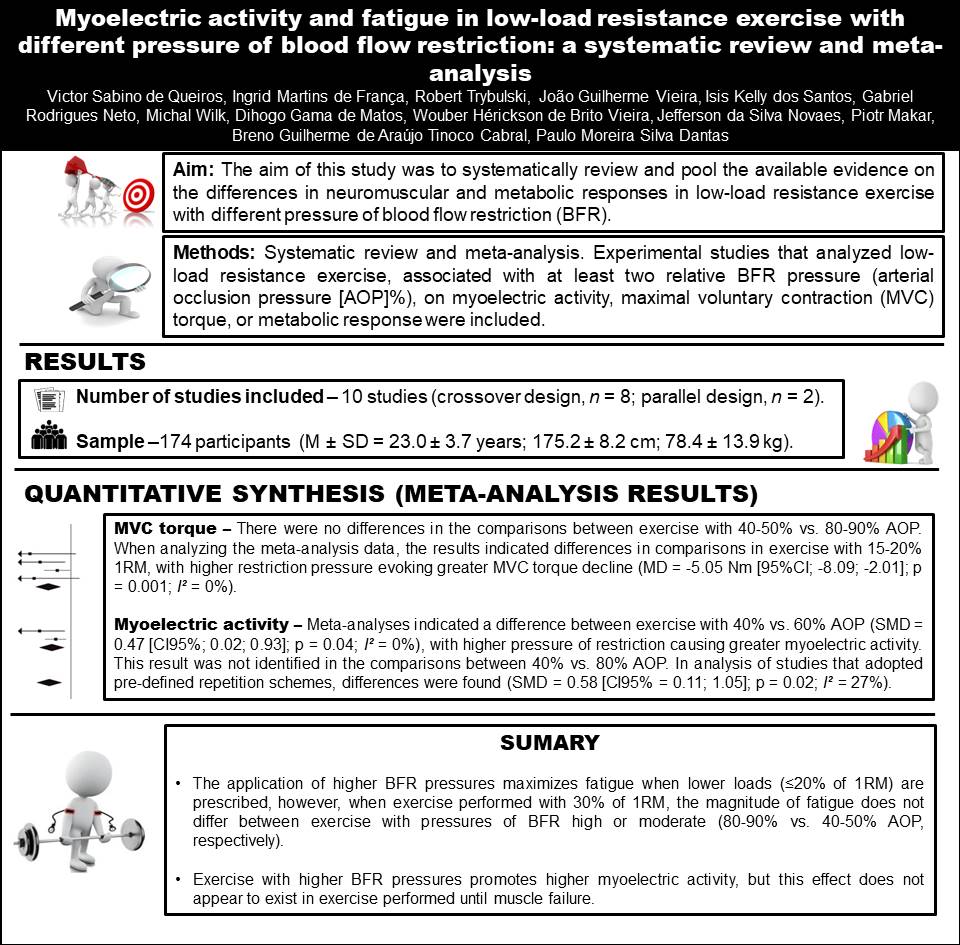
Supplementary Figures

**Supplementary Figure 1.** Graphic abstract.
